# Development of a *Cryptosporidium *oocyst assay using an automated fiber optic-based biosensor

**DOI:** 10.1186/1754-1611-1-3

**Published:** 2007-10-10

**Authors:** Marianne F Kramer, Graham Vesey, Natasha L Look, Ben R Herbert, Joyce M Simpson-Stroot, Daniel V Lim

**Affiliations:** 1 Division of Cell Biology, Microbiology, and Molecular Biology, Department of Biology, University of South Florida, 4202 E. Fowler Ave. SCA110, Tampa, FL, 33620 USA; 2 BTF Pty Ltd, P.O. Box 599, North Ryde BC, NSW 1670 Australia; 3 Faculty of Science, University of Technology Sydney, P.O. Box 123, Broadway, NSW 2007 Australia

## Abstract

An intestinal protozoan parasite, *Cryptosporidium parvum*, is a major cause of waterborne gastrointestinal disease worldwide. Detection of *Cryptosporidium *oocysts in potable water is a high priority for the water treatment industry to reduce potential outbreaks among the consumer populace. Anti-*Cryptosporidium *oocyst polyclonal and monoclonal antibodies were tested as capture and detection reagents for use in a fiber optic biosensor assay for the detection of *Cryptosporidium *oocysts. Antibodies were validated using enzyme-linked immunosorbent assays, flow cytometry, Western blotting and fluorescent microscopy. Oocysts could be detected at a concentration of 10^5 ^oocysts/ml when the polyclonal antibodies were used as the capture and detection reagents. When oocysts were boiled prior to detection, a ten-fold increase in sensitivity was achieved using the polyclonal antibody. Western blotting and immunofluorescence revealed that both the monoclonal and polyclonal antibodies recognize a large (>300 kDa) molecular weight mucin-like antigen present on the surface of the oocyst wall. The polyclonal antibody also reacted with a small (105 kDa) molecular weight antigen that was present in boiled samples of oocysts. Preliminary steps to design an in-line biosensor assay system have shown that oocysts would have to be concentrated from water samples and heat treated to allow detection by a biosensor assay.

## Background

*Cryptosporidium parvum *is an intestinal protozoan parasite that continues to be a major cause of waterborne gastrointestinal disease worldwide. Detection and removal of *Cryptosporidium *oocysts in potable water are high priorities for the water treatment industry to reduce potential outbreaks among the consumer populace. In the widely publicized 1993 Milwaukee *Cryptosporidium *outbreak, oocysts passed through the filtration system of one of the city's water treatment plants and an estimated 403,000 people suffered from gastroenteritis [1]. The breakdown of the filtration process was found to be related to high turbidity values of the water. The United States Environmental Protection Agency (EPA) regulatory approaches for *Cryptosporidium *removal in drinking water are currently based on filtration with compliance met using turbidity standards. Presently, detection of *Cryptosporidium *oocysts in water using conventional microbial analysis is labor intensive and can take days to complete [2,3]. Although water leaving a treatment plant may initially be safe for consumption, potable water supplies in the distribution system are vulnerable to intentional contamination. An extended analysis period could lead to large outbreaks of intestinal illness before either a breakthrough event or intentional contamination occurrence is detected. Therefore, there is a need for a rapid and automated assays targeted to detect potential pathogens in drinking water.

Members of the genus *Cryptosporidium *are intracellular coccidian parasites of mammals, birds, reptiles, and fish [4]. Upon ingestion by a suitable host, oocysts undergo excystation and release sporozoites into the intestine that then infect the microvillous border of the epithelial cell surfaces. Intracellular forms then undergo development and eventually produce sporulated oocysts that are passed from the host via feces. The intact oocyst is a thick-walled, double-layered structure containing four sporozoites and is resistant to chemical disinfectants including those used by many water purification processes; thus, filtration is the preferred method of removal [1,5].

For identification of breakthrough or intentional contamination events in the distribution system, a real-time or near-real-time detection system is needed that can operate continuously and autonomously. Many systems are in development for sampling and concentrating water from the supply lines; however, for any of them to be successful, they need to be coupled with biological detection systems such as biosensors. Biosensors have the potential to meet the need for rapid, sensitive, and versatile microbial detection systems. Fiber optic evanescent wave biosensors, in particular, have been used to detect a wide variety of molecules, including but not limited to fraction 1 antigen of *Yersinia pestis *[6], *Clostridium botulinum *toxin A [7], pseudexin and ricin toxin [8], trinitrotoluene (TNT), [9,10], polymerase chain reaction-amplified DNA [11], staphylococcal enterotoxin [12], and D-dimer [13]. In addition, fiber optic evanescent wave biosensors have been utilized to detect the human pathogens *Listeria monocytogenes *[14,15], *Salmonella enterica *[16], and *Escherichia coli *O157:H7 [17-20].

An automated evanescent wave biosensor (RAPTOR; Research International, Monroe, WA.) has been selected as an example for development of a *Cryptosporidium *assay. The RAPTOR utilizes an analysis coupon that is easily pre-treated to specifically target the pathogen of interest prior to inserting it into the biosensor for sample testing. The fiber optic biosensor assay is based on a sandwich immunoassay that utilizes antibodies or other molecules to capture the target pathogen from the sample matrix. The captured target pathogen is then tagged with a cyanine 5-labeled (Cy5) reporter antibody. A 635-nm laser diode provides the excitation light that is passed through the proximal end of each of the four injection-molded optical polystyrene fiber optic waveguides contained in the coupon. Fluorescent reporter antibodies within approximately 100–1000 nm of the waveguide surface are excited by the evanescent field, and a portion of their emission energy recouples into each fiber. A photodiode allows for quantitation of the collected emission light at wavelengths above 650 nm. Emission from the Cy5 is recorded in picoamperes (pA). The data are expressed as increases in fluorescence proportional in magnitude to the target pathogen concentration. Sample preparation is typically minimal since particulate matter does not interfere with the assay performance. The coupon can be reused for up to 20 different assays as long as negative assay results are obtained.

In this study, polyclonal and monoclonal anti-*Cryptosporidium *oocyst antibodies were used as capture and detection molecules to develop an assay for detection of *Cryptosporidium *oocysts using the automated evanescent waveguide-based biosensor. Potentially, the automated detection system could run autonomously in conjunction with an in-line concentration unit to continuously test for the presence of oocysts in a public potable water distribution system. A rapid automatic biosensor that effectively detects *Cryptosporidium *oocysts in water distribution systems downstream of treatment facilities would be highly desired by the water and public health industries.

This study focuses on problems and their solutions that were encountered in the development of a rapid automatic biosensor assay for detection of *Cryptosporidium *oocysts in drinking water lines. This system exemplifies the use of effective biology-based technologies (molecular biology and immunology) applied to a societal need for prevention of disease and bridges both biological and environmental engineering disciplines.

## Methods

### *Cryptosporidium parvum *oocysts and antibodies

*C. parvum *oocysts of Iowa isolate, bovine, from experimentally infected calves were purchased from Waterborne, Inc. (New Orleans, LA) or from the Sterling Parasitology Laboratory (University of Arizona, Tucson, AZ). Oocysts were stored in 0.1 M phosphate-buffered saline, pH 7.4 (PBS) at 4°C for no more than 6 weeks. Oocysts were serially diluted using PBS before testing in biosensor assays or enzyme-linked immunosorbent assays (ELISAs).

An IgG1 subclass monoclonal mouse antibody specific to the surface of *Cryptosporidium *oocyst walls [21] and a polyclonal rabbit antibody raised against the same antigen were obtained from BTF Pty Ltd (North Ryde NSW, Australia). Antibodies were tested as capture and detection molecules for use in ELISA and evanescent waveguide-based biosensor assays targeted to detect *Cryptosporidium *oocysts. For biosensor assays, antibodies were diluted, labeled with biotin or cyanine 5 (Cy5), and purified as previously described [19].

### Pre-treatment of oocysts

In many experiments, oocysts were treated prior to use in ELISAs, sodium-dodecyl-sulfate polyacrylamide gel electrophoresis (SDS-PAGE), or biosensor assays. Certain conditions such as addition of sodium taurocholate at 37°C have been reported to optimize excystation of isolates of *Cryptosporidium *in cell culture [22-25]. Treatments included the addition of sodium taurocholate (bile) or sodium dodecyl sulfate (SDS) at 5–10% final concentration and/or incubations at 37°, 50°, 60°, 75°, or 100°C (boiled) for 30 sec to 20 minutes and/or freeze-thawing the oocysts at -80°C six times. In some cases, oocysts boiled for 10 min were separated into a soluble fraction and an insoluble fraction by centrifuging treated oocysts for 10 min at 16,000 × g, 4°C, using an Eppendorf 5415R centrifuge (Brinkmann Instruments, Inc., Westbury, NY). The supernatant fluid was collected into a separate tube (soluble fraction) and the pellet was resuspended to the sample's original volume using PBS (insoluble fraction).

### Enzyme-linked immunosorbent assays (ELISAs)

Both the polyclonal and the monoclonal anti-*Cryptosporidium *oocyst antibodies, were evaluated for detection of *C. parvum *oocysts using ELISA. Volumes of 100 μl of all reactants were added to duplicate wells of 96-well microplates (Nunc MaxiSorp^®^, Nalge Nunc International, Rochester, NY). Serial dilutions of control or pre-treated oocysts were adsorbed to wells of plates for 18 h at 4°C. All further incubations were performed at 24°C. Wells were washed one time with PBS containing 0.1% Tween 20 (PBST) and blocked using blocking buffer (2 mg/ml casein, 2 mg/ml bovine serum albumin (BSA) in PBS). Plates were washed again, and dilutions of primary antibody (anti-*Cryptosporidium *oocyst monoclonal or polyclonal antibody) in blocking buffer were incubated in wells for 30 min. Wells were then washed three times with PBST, and horseradish peroxidase-labeled secondary antibody, anti-rabbit IgG or anti-mouse IgG (Kirkegaard & Perry Laboratories Inc., Gaithersburg MD) at a 1:1000 dilution in blocking buffer was added and incubated for 30 minutes. Wells were washed three times with PBST, and QuantaBlue reporter substrate was added (Pierce Biotechnology, Rockford, IL), followed by a 15 min incubation period. QuantaBlue stop solution was then added and fluorescence, detected as relative fluorescence units (RFU), was measured at 325 nm excitation and 420 nm emission using a Spectra Max Gemini XS fluorometer (Molecular Devices, Sunnyvale, CA). All ELISAs were performed at least two times using duplicate wells for each parameter tested. The signal to noise (S/N) ratio was calculated by dividing the mean RFU for the sample test wells by the negative control mean RFU. The negative control wells contained all additions except oocysts or primary antibody. Detection was considered positive when the S/N ratio was a minimum of 2.0.

### SDS-PAGE and Western blot

SDS-PAGE was conducted on 3–8% Tris-acetate gels (NuPage, Invitrogen Life Technologies, Carlsbad, CA) using Tris-Tricine running buffer (NuPage). Samples of boiled oocysts and control oocysts were loaded directly onto the gel or were reduced by 5 mM Tri-n-butyl phosphine (TBP, Sigma Aldrich, St. Louis, MO) before loading. After SDS-PAGE, gels were electrotransferred using a mini-Protean II blotter (BIORAD, Inc. Hercules, CA) to 0.22 μm pore size PVDF membranes (Millipore Corp., Bedford, MA) and membranes were blocked for 30 minutes at 24°C using 5% skim milk plus 5% bovine serum albumin (Sigma) in PBS. Membranes were incubated with either the anti-*Cryptosporidium *oocyst polyclonal or monoclonal antibody at a concentration of 5 μg/ml for 30 minutes at 24°C. After washing membranes with PBS, bound antibodies were detected using 0.5 μg/ml alkaline phosphatase conjugated goat anti-rabbit IgG or alkaline phosphatase conjugated sheep anti-mouse IgG (Sigma) incubated for 30 min at 24°C. After washing, membranes were developed using Sigma FAST™ 5-bromo-4-chloro-3-indolyl phosphate/nitro blue tetrazolium according to the manufacturer's instructions.

### Fluorescent microscopy

To avoid exposing oocysts to heat, oocysts were labeled with polyclonal antibody using the following filtration method. Volumes of 100 μl of all reactants were added to 96-well filter plates (Mulitscreen-BV, 1.2 μm hydrophilic low protein binding Durapore membrane; Millipore Corp.). All incubations were performed at 24°C. Control or boiled oocysts were added to wells, and vacuum was applied to remove liquid. Blocking buffer was added to oocysts in wells, and primary antibody (anti-*Cryptosporidium *oocyst polyclonal antibody, 5 μg/ml) was added and incubated for 30 min. Vacuum was applied to the wells and the wells were then washed three times with PBS.

Fluorescein-5-isothiocyanate (FITC)-labeled secondary antibody, anti-rabbit IgG (Sigma) at 1:500 dilution in blocking buffer, was added and incubated for 30 min. Oocysts were also prepared without primary antibody as a negative control. Vacuum was again applied to the wells, and wells were washed three times with PBS. Oocysts were then resuspended in PBS, added to microscope slides and covered with coverslips. In addition, samples of boiled, freeze thawed and excysted oocysts were immunofluorescently labeled with polyclonal and monoclonal antibody by mixing with FITC labeled polyclonal or monoclonal antibody at a concentration of 5 μg/ml in PBS plus 1% BSA and leaving at 24°C for 15 min. A drop of the stained samples was placed onto microscope slides and a coverslip applied. The oocysts were viewed under light and epifluorescence microscopy (Olympus BX 60, Olympus America, Inc., Lake Success, NY) using a U-MWIBA filter. Photographs were taken at 400× magnification with an Olympus Q Color 3 cooled camera. Image contrast was adjusted using picture brightness/contrast feature of Microsoft Word program to darken field background in order to visualize cells.

### Flow cytometry

Samples (50 μl) of boiled and control oocysts were mixed with 100 μl aliquots of concentrated filter backwash water from a water treatment plant. The backwash water sample was prepared by collecting 20 L of water from the backwash cycle of a filter bed at a local treatment plant (Richmond, Sydney). The sample was allowed to settle for 48 h at 4°C and supernatant fluid carefully removed. The remaining 1 L volume was stored at 4°C until used. The backwash concentrate was added to simulate the background that might be encountered with testing of a real water sample. The samples were mixed with the monoclonal or polyclonal antibody that had been conjugated with fluorescein isothiocynate as described previously [26] at a final concentration of 10 μg/ml in PBS plus 0.5% (w/v) bovine serum albumin. After 10 minutes incubation at 24°C, the samples were analyzed using a Becton Dickinson FACScalibur flow cytometer. The threshold was set on green fluorescence (FL1) and the entire sample was analyzed. Data were displayed on a scatter plot of FL1 (y axis) and forward scatter (x axis).

### Biosensor instrument and waveguide preparation

The RAPTOR Plus 4S (RAPTOR) is a portable, automatic fiber optic biosensor manufactured by Research International (Monroe, WA). Polystyrene waveguides, 4.5 cm in length, used with the RAPTOR and produced by Research International, were sonicated for 30 sec in an isopropanol bath. Waveguides were rinsed with deionized water; the distal tip of the waveguide was dipped in black paint to provide a light dump. After the paint dried, waveguides were added to glass capillary tube-incubation chambers and were incubated at 4°C for 18 to 22 h with 100 μg/ml streptavidin (Sigma). Waveguides were rinsed with PBST, added to clean glass capillary tube-incubation chambers and incubated with 100 μl of 100 μg/ml biotinylated antibody in PBS (capture antibody) at 24°C for 30 min. The capture antibody solution was replaced with fresh solution and incubated an additional 30 min. Four waveguides coated with capture antibody were then glued into each RAPTOR coupon which was sealed with sealing tape and label added. The RAPTOR was programmed to run automatically using a start-up recipe and a sample recipe. The start-up recipe was used at the beginning of each experiment and consisted of the following steps. The four waveguides were rinsed with an equivalent of 2 ml of PBST two times. The 635 nm diode laser was then activated, and the pA signal was recorded after 5 sec. The sample recipe was used to take baseline (blanks) and sample readings and consisted of incubating each of the four waveguides with a different 0.2 ml sample for five min, followed by incubating each waveguide with an additional 0.2 ml sample for five min. The waveguides were then rinsed with PBST; detection antibody was added and incubated for five min. The detection antibody was then returned to the holding vessel via the internal pump system. Waveguides were rinsed twice with PBST. The 635 nm diode laser was activated while PBST was sitting in the coupon channels, and the pA signal was recorded after 5 sec. PBST was then discarded to waste. All automated assays were carried out at 24°C.

### Baseline readings

Six baseline readings were taken for each coupon tested. Detection antibody consisted of Cy5-labeled monoclonal anti-*Cryptosporidium *oocyst antibody, 10–20 μg/ml in detection buffer (2 mg/ml casein, 2 mg/ml bovine serum albumin in PBS). The pA value for each baseline reading was subtracted from the subsequent baseline reading; the calculated value was designated as the ΔpA previous signal for baselines 2 through 6. The detection limit was calculated as the mean ΔpA previous signal for baselines 2 through 6 (mean baseline value) plus three times the standard deviation.

### Biosensor sensitivity assay

A sample of PBS with or without *Cryptosporidium *oocysts in a volume of 0.4 ml was added to four 1.5 ml tubes. Sample tubing from the RAPTOR was inserted into each of the 4 sample tubes, and the "run assay" button was pushed. The RAPTOR automatically ran a sample recipe as described previously. When the first sample was tested, the last baseline reading was subtracted from the sample reading. When additional samples were tested, each previous sample reading was subtracted from the next sample reading. These calculated values for the samples were designated as the change in pA from previous signal for each sample. At least two waveguides were tested in each assay, and at least triplicate samples for each oocyst concentration were tested on each waveguide. All assays were performed at least two times.

## Results

### Effect of oocyst pre-treatment on antibody binding

**Figure 1:**
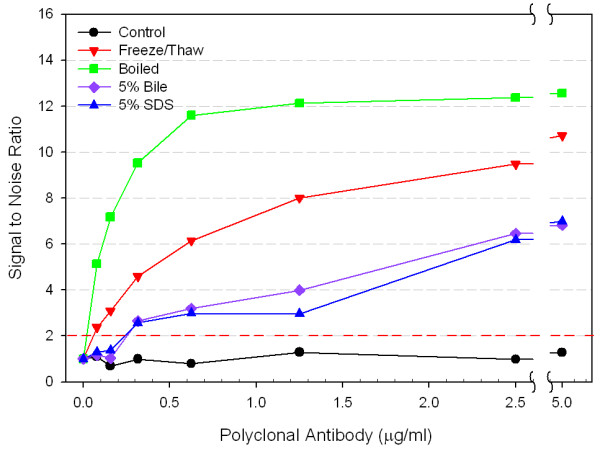
**Effect of *C. parvum *oocyst pre-treatments on the binding of polyclonal anti-*Cryptosporidium *antibody as determined by ELISA**. Oocysts (10^5 ^oocysts/well) were tested as control, frozen and thawed six times, boiled for 10 minutes, or incubated at 37°C in 5% bile or in 5% SDS. Error bars represent one standard deviation and fall within the symbols for each point. Signal to noise values above 2 are considered positive. The standard deviation for the noise (PBS control wells) was 0.07.

The anti-*Cryptosporidium *polyclonal antibody was first evaluated for detection of *Cryptosporidium *oocysts using ELISA. Oocysts tested were either control or boiled, freeze/thawed, or incubated with 5% bile or SDS at 37°C. At least a five-fold higher S/N ratio was obtained when the polyclonal antibody was bound to boiled or freeze/thawed treated oocysts as compared to control (Figure 1). Treatment of oocysts with bile or SDS also improved the antibody binding affinity; S/N ratios were approximately three-fold higher than those for untreated oocysts. Shorter boiling times as low as two minutes gave similar results to the ten minute treatment (data not shown). Lower temperature treatments of 50°, 60°, 75°C for 10 minutes also improved antibody binding to oocysts by 1.5-, 2.4-, and 4-fold, respectively, suggesting that oocyst heat treatment increases antibody binding to the oocysts. A 10 minute incubation at 37°C gave similar ELISA results compared with the untreated control oocysts (data not shown).

**Figure 2:**
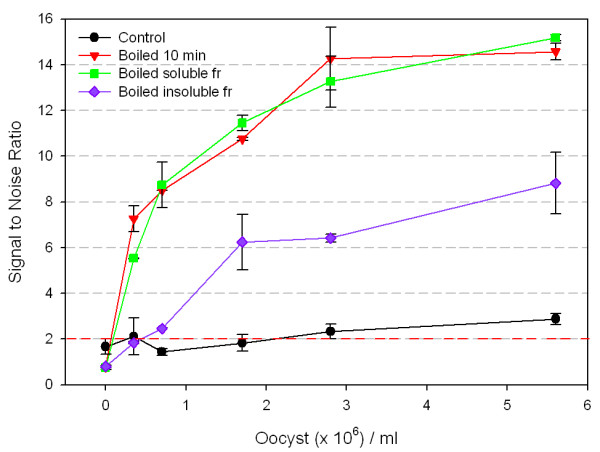
**ELISA detection of soluble and insoluble antigens of boiled *C. parvum *oocysts**. Oocysts were control, boiled for 10 minutes, or boiled 10 minutes and separated into insoluble fractions and soluble fractions by centrifugation. Oocysts were then detected using anti-*Cryptosporidium *polyclonal antibody (1.25 μg/ml). Error bars represent one standard deviation. Signal to noise values above 2 are considered positive.

Boiled oocysts were then divided into soluble and insoluble fractions by boiling the oocysts, centrifuging and collecting the supernatant fluid (soluble fraction) and resuspending the centrifuged pellet in PBS (insoluble fraction). The aggregate boiled sample and soluble fraction produced similar S/N ratios and were at least four times higher than that of the unboiled control. (Figure 2). The S/N ratio for the insoluble fraction was three-fold higher than the control, but half that of the whole or soluble portion of the sample.

**Figure 3:**
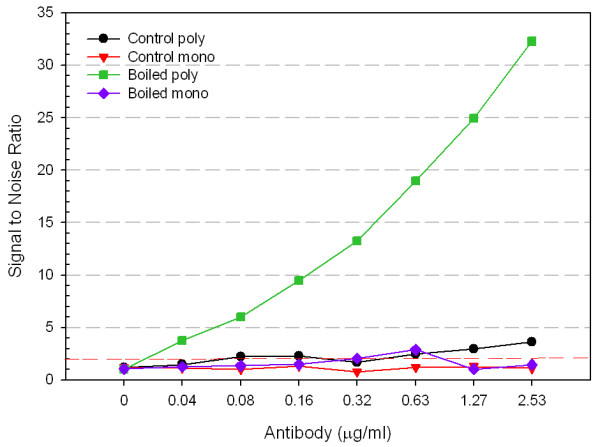
**Monoclonal and polyclonal anti-*Cryptosporidium *antibody binding to boiled *C. parvum *oocyst antigens**. Oocysts (10^5 ^oocysts/well) were held as control or boiled for 10 minutes and then analyzed using polyclonal antibody or monoclonal antibody in an ELISA: control poly; control mono; boiled poly; boiled mono. Error bars represent one standard deviation and fall within the symbols for each point. Signal to noise values above 2 are considered positive. The standard deviation for the noise (PBS control wells) was 0.10 and 0.07 for the polyclonal and monoclonal antibodies, respectively.

The monoclonal antibody was then tested using ELISA as described to determine whether boiled oocysts gave higher S/N ratios as previously observed for the polyclonal (Figure 3). In contrast to results obtained with polyclonal antibody, the monoclonal antibody produced no significant difference in S/N ratios for boiled oocysts as compared to control. The result was the same irrespective of antibody concentration tested.

### *C. parvum *oocyst antigen detection by polyclonal antibody

**Figure 4:**
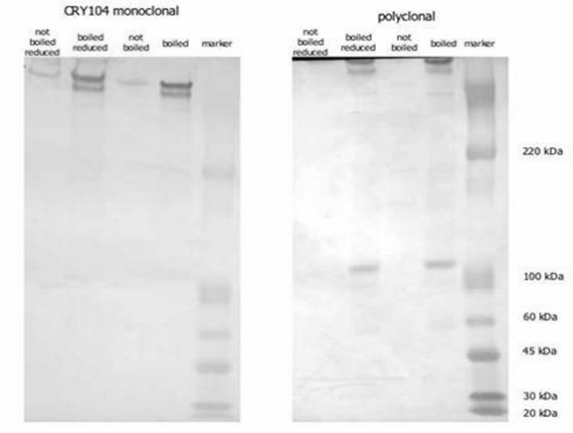
**Analysis of *Cryptosporidium *oocyst antigens by Western blotting**. Samples of boiled/reduced and control oocysts were electrophoresed by SDS-PAGE and the blotted membranes were probed with either polyclonal or monoclonal anti-*Cryptosporidium *FITC labeled antibody.

A Western blot of oocysts was performed in order to determine whether the polyclonal antibody was binding to different antigens in soluble and insoluble oocyst fractions. Both the polyclonal and monoclonal antibodies recognized a large (>300 kDa) molecular weight antigen that appeared as two bands in the boiled oocyst samples (Figure 4). The polyclonal antibody also reacted with a 105 kDa antigen from the soluble fraction of the boiled oocysts. The monoclonal antibody did not react with the 105 kDa molecular weight antigen. Results were similar for both reduced and non-reduced samples.

### Antibody binding analysis by flow cytometry

**Figure 5:**
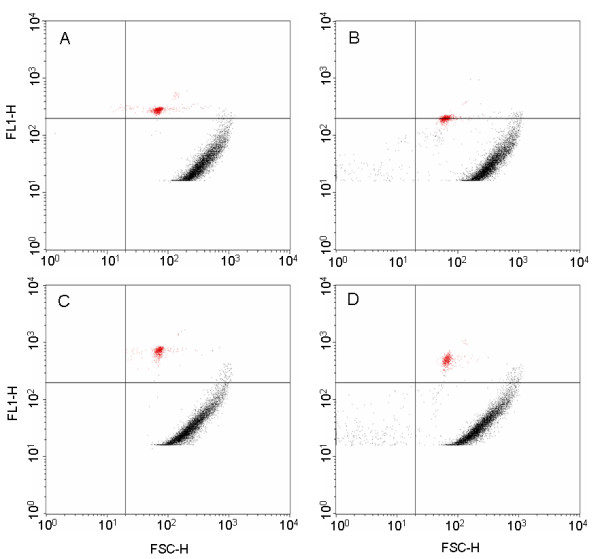
**Analysis of polyclonal and monoclonal antibody binding to boiled and unboiled *C. parvum *oocysts using flow cytometry**. Particle size (x-axis) vs fluorescence (y-axis) is depicted by the forward scatter. The top plots present polyclonal antibody binding to either control (A) or boiled (B) oocysts and the bottom plots present monoclonal antibody binding to either control (C) or boiled (D) oocysts. The small red population represents the fluorescent oocysts and the larger curved population represents debris particles present in the water sample.

Analysis of control and boiled oocysts that had been stained with FITC labeled monoclonal or FITC labeled polyclonal antibody demonstrated a slight decrease in fluorescence after boiling (Figure 5). The separation between the oocysts and the debris particles represented the S/N ratio for this type of analysis. The S/N ratio did not change notably with any of the samples.

### Antibody binding analysis by fluorescent microscopy

**Figure 6:**
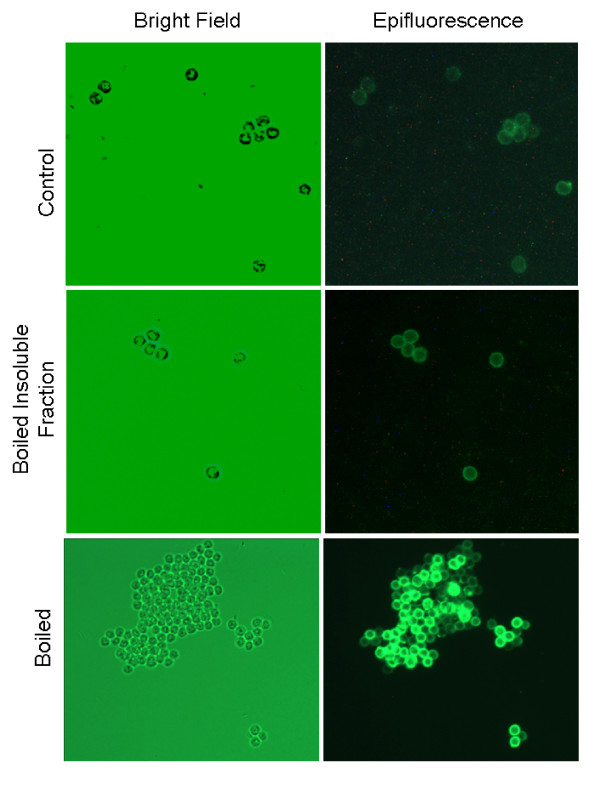
**Epifluorescent images of polyclonal anti-*Cryptosporidiu *m antibody binding to oocyst walls**. Oocysts were labeled with rabbit polyclonal antibody, which was then tagged with FITC-labeled anti-rabbit IgG. Oocysts were viewed and imaged using bright field and an Olympus UM-WIBA FITC fluorescence filter (400× magnification).

Boiling and other oocyst treatments could possibly release internal antigens from the oocysts that are recognized by the polyclonal antibody. This release could be causing the increase in ELISA S/N ratio with the polyclonal antibody. Fluorescent microscopy was used to examine samples of control, boiled, freeze thawed and excysted oocysts. Uniform fluorescence of the oocyst wall with no internal fluorescence was observed in all samples (data not shown for freeze thawed and excysted oocysts). The boiled oocyst sample appeared slightly more fluorescent than the other samples (Figure 6).

### Biosensor assays

**Figure 7:**
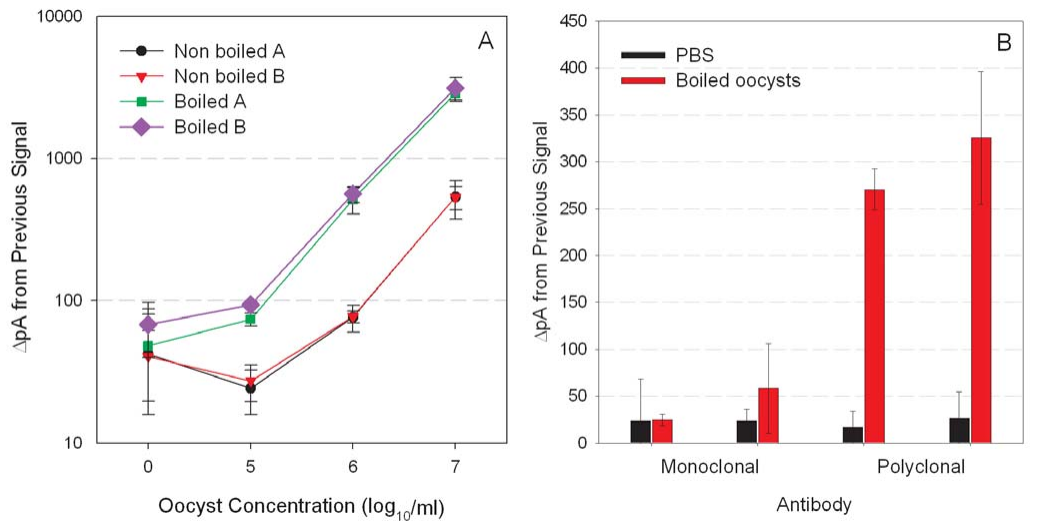
**Fiber optic biosensor assays for the detection of *Cryptosporidium parvum *oocysts**. (A) Representative biosensor results using Cy5-labeled polyclonal antibody comparing signals obtained for non-boiled (control) or boiled oocysts. Replicates designated by A and B identifiers. (B) Biosensor assay using either PBS or boiled oocysts (10^6 ^oocysts/ml) to compare Cy5-labeled monoclonal or polyclonal antibodies. Error bars represent ± one SD for the mean of three assays from each waveguide.

For each experiment four samples of oocysts suspended in PBS were automatically injected into a coupon prepared with polyclonal anti-*Cryptosporidium *antibodies as the capture molecule. For detection, either Cy5-labeled polyclonal or monoclonal detector antibodies were used. The average detection limits for waveguides tested using polyclonal antibody and monoclonal antibody were 89 and 110, respectively. Values for ΔpA from previous signal that are above the detection limit are considered positive for the detection of *Cryptosporidium *oocysts. When polyclonal antibody was used as both the capture and detector molecule, boiled oocysts were detected at 10^5 ^oocysts/ml concentrations, whereas control oocysts (not boiled) were detected at 10^6 ^oocysts/ml concentrations (Figure 7A). When the monoclonal antibody was used as the detector molecule, the lowest concentration of boiled oocysts detected was 10^6 ^oocysts/ml (Figure 7B), but the values for the change in pA from the previous signal for detection of 10^6 ^oocysts/ml using the Cy5-labeled monoclonal antibody were not significantly higher than the signals obtained with samples containing only PBS.

## Discussion

As reported waterborne outbreaks of cryptosporidiosis have increased over the last few years, and as new regulations have been implemented, interest in detecting *Cryptosporidium *in water has grown. Many articles have been written that focus on various methods for detecting oocysts in water (see reviews [27,28]), yet most if not all deal with methods that rely upon grab samples, without the potential for advancing to in-line detection. Development of an assay to be used on a biosensor is the initial step to producing an automated system for in-line detection of *Cryptosporidium *oocysts in potable water. As described herein, a RAPTOR biosensor was used to develop an assay targeted to detect *Cryptosporidium *oocysts in water as a step towards producing an autonomous system.

As polyclonal antibodies are known to be effective capture molecules, and monoclonal antibodies have been widely used as reporter molecules, they were produced by a patented technique that involves immunizing mice or rabbits with semi-solubilized oocyst wall antigen [21] and the resulting anti-*Cryptosporidium *oocyst antibodies were evaluated. Because bile treatment is frequently used to optimize excystation of oocysts [22-25], bile was tested in addition to SDS to try to improve S/N ratios. Treatment of oocysts with bile or SDS improved the S/N ratio as compared to controls but not as significantly as temperature treatments. The higher ratios obtained for boiled oocysts as compared to those treated with bile or SDS suggested that oocyst excystation alone was not responsible for the improved antibody binding. Boiling the oocysts may release additional immunogenic proteins from the oocysts or may denature or degrade proteins so that they are more readily accessible for polyclonal antibody binding, thereby producing a higher signal.

Flow cytometry is commonly used as a preliminary purification/concentration step and typically presumptive oocysts are sorted out of the water and confirmed by manual microscopy [29]. When the polyclonal antibody was tested using flow cytometry, control oocysts generated greater fluorescence signals than boiled oocysts. Detection using flow cytometry is based on size and, thereby, solubilized analytes released by boiling were excluded from detection. In ELISA assays, oocysts and any associated free antigens were adsorbed to the bottom of wells in 96-well plates. Therefore, antibody could bind to any or all antigens present or possibly adsorbed to the plastic, including solubilized antigens, and a greater signal is produced. To test this hypothesis, boiled oocysts were divided into soluble and insoluble fractions and retested. ELISAs showed that the polyclonal antibody did bind to soluble antigens found in the soluble fraction. Boiling and freeze/thawing most likely disassociated antigens from the oocyst, thereby allowing antibodies to have greater access to oocyst antigens. These soluble antigens could be detected using ELISA, but could not be detected using flow cytometry.

Analysis by Western blotting revealed that both the polyclonal and monoclonal anti-*Cryptosporidium *antibodies recognize a large (>300 kDa) antigen. Analysis of the soluble fraction of boiled oocysts revealed that a small (105 kDa) antigen was released by boiling the oocysts. This antigen was recognized by the polyclonal antibody but not by the monoclonal antibody. It is unknown whether this small antigen is released from the boiled oocysts or is a fragment of the larger antigen but its presence in the soluble fraction may account for the differences in signal between ELISA and flow cytometry analysis.

The fiber optic biosensor only excites the fluorophore of fluorescently tagged reporter antibodies present within the evanescent field generated 100 nm to 1000 nm from the surface of the fiber optic waveguide [30,31]. The fluorescently tagged antibodies binding to the majority of antigens on the surface of the unboiled larger oocysts (4–6 μm diameter) are not within the evanescent field and thus are not excited. Only the portion bound to the oocysts closest to the surface are detected by the biosensor, which results in poorer sensitivity. The smaller soluble analytes generated during oocyst boiling, as shown by Western blot, are more likely to be located within the evanescent field of the biosensor due to their small size. When these smaller analytes are bound by Cy5-labeled detection antibodies, the Cy5 is excited, and the smaller analytes are detected by the biosensor in addition to the bound oocysts, thus increasing signal. The larger portion of the Cy5 antibodies excited by the evanescent field results in an increase in pA signal and ultimately an increase in sensitivity.

Flow cytometry and fluorescence microscopy revealed that not all the antigen was removed from the oocyst surface by boiling. The boiled oocysts were slightly more fluorescent than the control oocysts and they were considerably more fluorescent than the debris particles present in the filter backwash water. This increase in fluorescence indicates that more antigen was accessible for binding although it was still adhered to the surface. This difference would suggest that the sensitivity of the biosensor could be increased considerably if a method for complete removal of the oocyst antigen prior to detection were developed. Complete removal of the antigen would allow for better binding due to size differential and possibly more complete coverage of available binding surface area.

In addition, the results demonstrate that flow cytometry is not always a suitable tool for screening antibodies, particularly if antibodies are being screened for other applications (e.g., capture molecules on biosensor). With flow cytometry, the S/N ratio was significantly higher when monoclonal antibody was bound to control oocysts than when polyclonal antibody was bound to both control and treated oocysts. This is in direct contrast to results obtained by ELISA method and biosensor assay, which showed the highest S/N ratio for boiled oocysts bound to polyclonal antibody. These results reaffirm that ELISA is a suitable method for evaluating antibodies for use in the biosensor assay.

The biosensor assay described herein is a first step towards an automated detection system for *Cryptosporidium *in water distribution systems. The results indicate that although current biosensor detection limits are high as compared to EPA regulations, there are ways of increasing sensitivity. The infectivity of *C. parvum *oocysts has been investigated in healthy adults and, although *C. parvum *isolates have been shown to differ in their infectivity, as few as 9 oocysts were reported to produce infection in 50% of individuals [32,33]. The *Cryptosporidium *biosensor assay using polyclonal antibody was capable of detecting boiled oocysts at 10^6^/ml concentration. Oocysts can occur in drinking water at minimum infectious dose levels (approximately 1 oocyst/ml) and, therefore, would not be detected using the described assay. However, appending the biosensor to an upstream concentration/pretreatment step would allow detection at clinically relevant levels. To develop an automated in-line biosensor for oocyst detection, a very simple oocyst treatment to optimize antibody binding would be required. A high temperature oocyst treatment could easily be incorporated and automated in conjunction with a concentrator. The concentrator would increase the number of oocysts present in a sample, followed by an in-line heat exchanger to treat the concentrated oocysts before being presented to the biosensor for testing. Unfortunately, the high temperature treatment would destroy oocyst viability, thereby preventing recovery and culture of oocysts from the waveguide for confirmatory testing. Therefore, each time a positive result is obtained from the biosensor, an aliquot of the pre-boiled sample would have to be collected and stored for confirmation. Development of an automated, in-line concentration and detection system for microorganisms is presently in progress. The system in development could easily be adapted for concentration and heating of oocysts, followed by oocyst detection using the biosensor assay.
